# DSGOST inhibits tumor growth by blocking VEGF/VEGFR2-activated angiogenesis

**DOI:** 10.18632/oncotarget.7982

**Published:** 2016-03-08

**Authors:** Hyeong Sim Choi, Kangwook Lee, Min Kyoung Kim, Kang Min Lee, Yong Cheol Shin, Sung-Gook Cho, Seong-Gyu Ko

**Affiliations:** ^1^ Department of Science in Korean Medicine, Graduate School, Kyung Hee University, 1 Hoegi, Seoul 130-701, Korea; ^2^ Department of Preventive Medicine, College of Korean Medicine, Kyung Hee University, 1 Hoegi, Seoul 130-701, Korea; ^3^ Department of Biotechnology, Korea National University of Transportation, Jeungpyeong, Chungbuk 368-701, Korea

**Keywords:** DSGOST, angiogenesis, tumor, VEGF, herbal medicine

## Abstract

Tumor growth requires a process called angiogenesis, a new blood vessel formation from pre-existing vessels, as newly formed vessels provide tumor cells with oxygen and nutrition. Danggui-Sayuk-Ga-Osuyu-Saenggang-Tang (DSGOST), one of traditional Chinese medicines, has been widely used in treatment of vessel diseases including Raynaud's syndrome in Northeast Asian countries including China, Japan and Korea. Therefore, we hypothesized that DSGOST might inhibit tumor growth by targeting newly formed vessels on the basis of its historical prescription. Here, we demonstrate that DSGOST inhibits tumor growth by inhibiting VEGF-induced angiogenesis. DSGOST inhibited VEGF-induced angiogenic abilities of endothelial cells *in vitro* and *in vivo*, which resulted from its inhibition of VEGF/VEGFR2 interaction. Furthermore, DSGOST attenuated pancreatic tumor growth *in vivo* by reducing angiogenic vessel numbers, while not affecting pancreatic tumor cell viability. Thus, our data conclude that DSGOST inhibits VEGF-induced tumor angiogenesis, suggesting a new indication for DSGOST in treatment of cancer.

## INTRODUCTION

Tumor angiogenesis plays an important role in tumor growth, as tumor vessels abundantly provide tumor cells with both nutrition and oxygen [1–4]. Furthermore, tumor cells metastasize to distant organs through tumor angiogenic vessels [3, 4]. Therefore, targeting tumor angiogenesis is one of crucial ways for cancer treatment. Vascular endothelial growth factor (VEGF) secreted from tumor cells works as tumor angiogenic environmental cue, as it promotes angiogenic abilities of endothelial cells expressing its endogenous receptor, VEGFR2 [3–6]. VEGF binds to VEGFR1 or VEGFR2 which there are able to form both homodimers and heterodimers between them in endothelial cells [6]. However, VEGFR2 is higher abundant than VEGFR1 on the endothelial cell surface [7, 8]. In addition, the pro-angiogenic signaling function of VEGFR1/2 heterodimer is not clearly deciphered [6, 7]. It is well defined that VEGF-activated VEGFR2 induces multiple intracellular signaling pathways including NF-κB signaling pathway [6, 9]. Therefore, VEGF-dependent intracellular signaling pathways are one of useful readouts for tumor angiogenesis.

There is a growing interest in the medicinal use of traditional Chinese medicines (TCMs) [10–13]. Danggui-Sayuk-Ga-Osuyu-Saenggang-Tang (DSGOST; Danggui-Sini-Jia-Wuzhuyu-Shengjian-Tang in Chinese, Tokishigyakukagoshuyushokyoto in Japanese) has long been used in treatment of vascular diseases including Raynaud's syndrome [14–18]. Our previous study first identified the molecular and cellular effect of DSGOST in Raynaud's syndrome [19]. In that study, we found that DSGOST inhibited endothelial cell contraction. Accordingly, we hypothesized that DSGOST might target tumor angiogenic vessels.

In this study, we investigated whether DSGOST inhibits angiogenesis, especially VEGF-induced tumor angiogenesis. DSGOST suppressed VEGF-induced endothelial cell migration, tube formation and invasion without affecting cell growth. In addition, DSGOST inhibited VEGF-induced intracellular angiogenic signaling in the endothelial cells. Our *in vivo* studies confirmed that DSGOST effectively suppressed VEGF-induced vascular leakage and angiogenesis [20]. Consistently, DSGOST inhibited *in vivo* xenograft mouse tumor growth by reducing angiogenic vessel numbers. Therefore, our study first identifies its effect in tumor angiogenesis and provides a new indication.

## RESULTS

### DSGOST inhibits VEGF-dependent endothelial cell migration, invasion and tube formation without affecting cell growth

**Figure 1:**
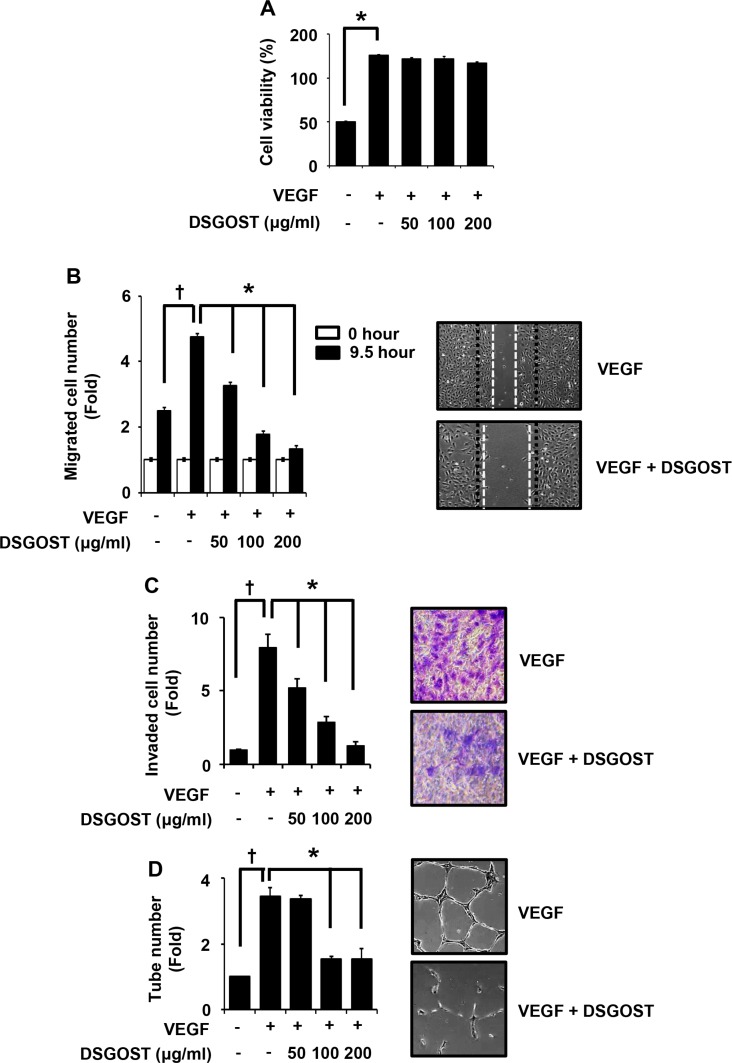
DSGOST inhibition of VEGF-induced angiogenic abilities *in vitro* **(A)** The effect of DSGOST on the viability in HUVECs was determined by the MTT assay (mean ± SD; *n* = 6). *P = 0.004 versus untreated cells. **(B)** Cell migration. DSGOST at the indicated concentrations inhibits VEGF-induced migration in cell scratching assays (left). ^†^*P* = 0.004 versus untreated cells and *P = 0.0001, 0.0003, 0.0001 versus only VEGF-treated cells. Representative images of cell migration results (right). **(C)** Cell invasion. DSGOST at the indicated concentrations inhibited VEGF-induced cell invasiveness in two-chamber assays (left). ^†^*P* = 0.0003 versus untreated cells and *P = 0.0002, 0.0003, 0.0001 versus only VEGF-treated cells. Representative images of cell invasion results (right). **(D)** Tube formation. DSGOST inhibits VEGF-induced tube formation (left). ^†^*P* = 0.0004 versus untreated cells and *P = 0.296, 0.0002, 0.0003 versus only VEGF-treated cells. The representative images of tube formation results (right).

We first examined DSGOST effect on VEGF-dependent *in vitro* angiogenic abilities of endothelial cells. Human umbilical vascular endothelial cells (HUVECs) were treated with 50 ng/ml of VEGF and different concentrations of DSGOST (50, 100 or 200 μg/ml) for 72 hours and then subjected to MTT assays. VEGF alone markedly induced the proliferation (*P* = 0.004), DSGOST did not affect VEGF-induced proliferation of HUVECs (Figure 1A). However, DSGOST inhibited VEGF-dependent migration of HUVECs in scratching assays, when cells were treated with VEGF and DSGOST for 9.5 hours (Figure 1B). Likewise, DSGOST repressed VEGF-dependent invasion of HUVECs in two chamber assays, when cells were seeded on matrigel-precoated top chamber and then treated with VEGF and DSGOST for 5 days (Figure 1C). In tube formation assays, DSGOST at 100 μg/ml inhibited VEGF-dependent tube formation by approximately 60%, when cells were treated with VEGF and DSGOST for 9 hours (Figure 1D). Therefore, our data indicate that DSGOST inhibits VEGF-dependent migration, invasion and tube formation of HUVECs *in vitro* without affecting cell proliferation. To confirm its effect on endothelial cells, we also examined its inhibitory effect in human dermal microvascular endothelial cells (HDMECs). DSGOST effect on HDMECs was quite similar to that on HUVECs, suggesting that DSGOST efficiently inhibits VEGF-induced angiogenic abilities of the endothelial cells.

### DSGOST inhibits angiogenic signaling by blocking VEGF binding to VEGFR2

**Figure 2:**
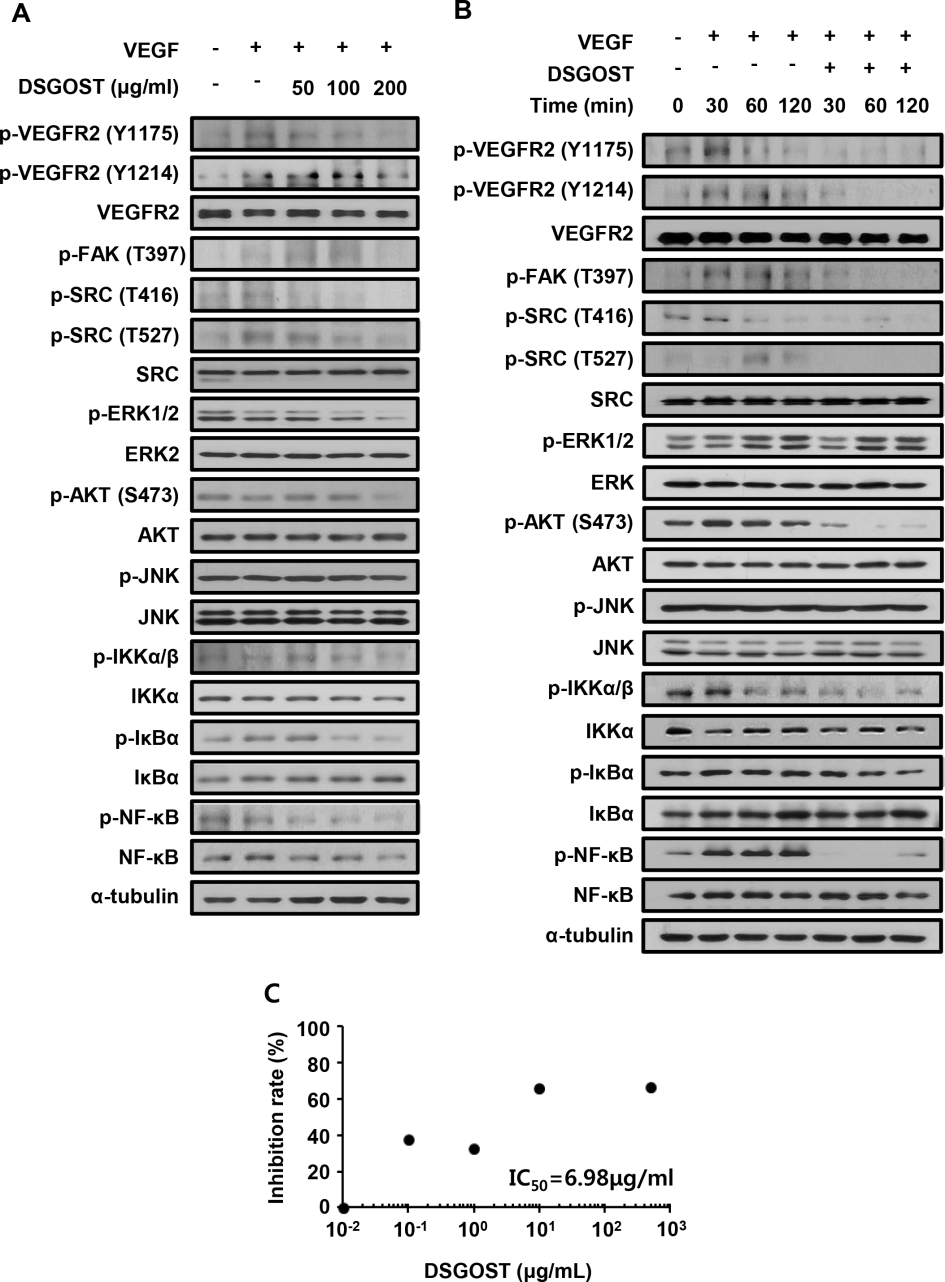
DSGOST affects VEGF-induced intracellular signaling **(A)** HUVECs were pretreated with DSGOST at different concentrations for 60 minutes and then treated with VEGF (50 ng/ml) for another 60 minutes. **(B)** Cells were treated with VEGF and DSGOST for the indicated time points. **(C)** DSGOST and btVEGF were treated on the plate where recombinant human VEGFR2 was coated.

We further examined DSGOST effect on VEGF-dependent intracellular signaling in endothelial cells. HUVECs were pretreated with different concentrations of DSGOST for 60 minutes and then treated with VEGF at 50 ng/ml for another 60 minutes. DSGOST inhibited phosphorylation of VEGF-induced intracellular signaling pathway molecules including VEGFR2 (Figure 2A). Moreover, when DSGOST inhibitory effect was examined for 120 minutes, DSGOST at 100 μg/ml inhibited VEGF-dependent phosphorylation of VEGFR2, FAK, SRC, AKT, IKKα/β, IκBα, and NF-κB (Figure 2B). Moreover, in our *in vitro* solid-phase binding assays, DSGOST blocked biotinylated VEGF binding to recombinant human VEGFR2 (Figure 2C). Thus, our data suggest that DSGOST inhibits angiogenic signaling by directly blocking VEGF binding to VEGFR2.

### DSGOST inhibits NF-κB-dependent MMP9 expression in VEGF-stimulated endothelial cells

**Figure 3:**
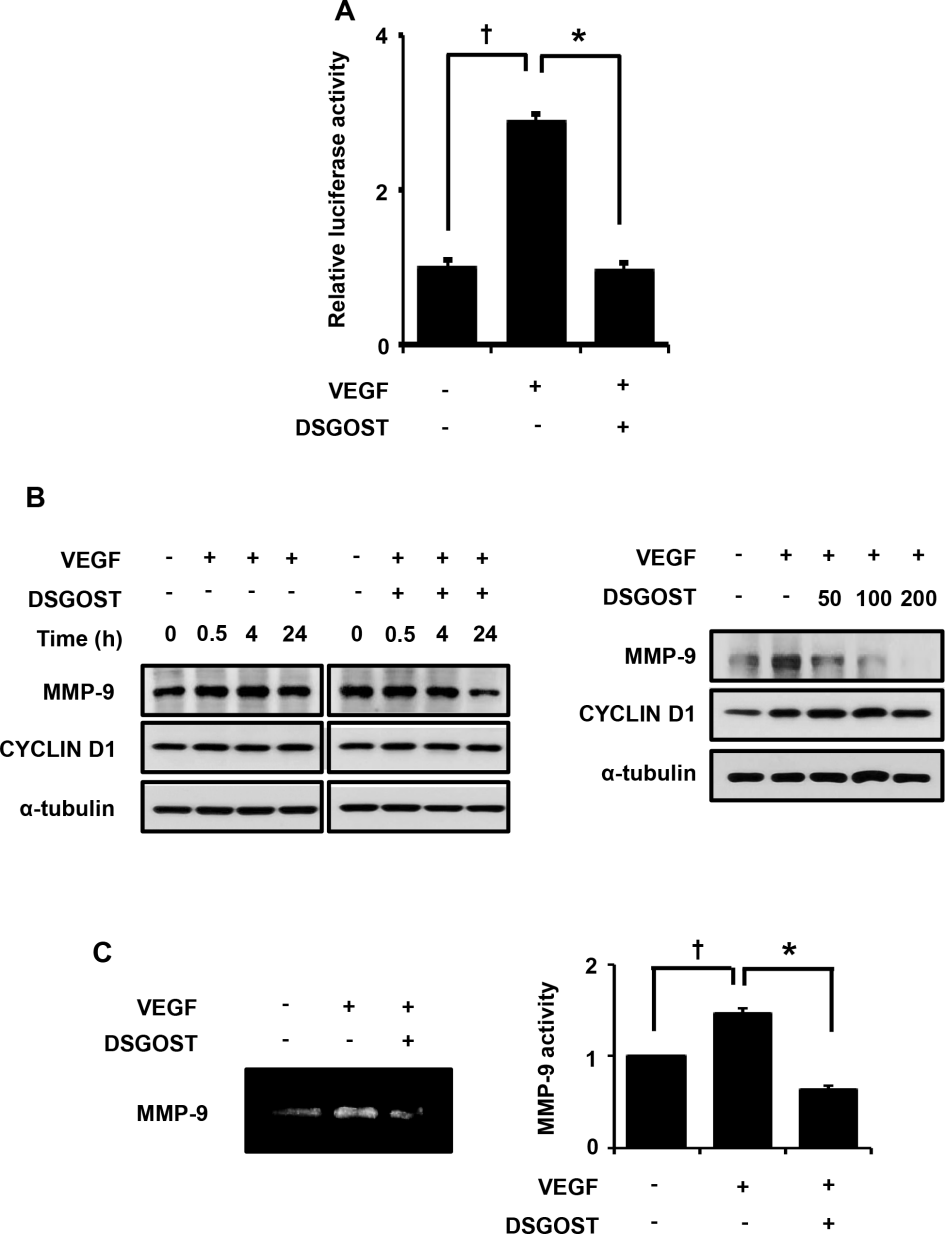
DSGOST inhibits VEGF activation of NF-κB signaling **(A)** NF-κB activity was measured using reporter gene assay (mean ± SD; *n* = 3). ^†^*P* = 0.007 versus untreated cells and *P = 0.008 versus only VEGF-treated cells. **(B)** Left panel, cells were treated with VEGF and DSGOST for the indicated time points and then MMP-9 expression pattern was analyzed. Right panel, cells were treated with VEGF and DSGOST at different concentrations for 24 hours. **(C)** MMP-9 activity was evaluated via zymography assay. ^†^*P* = 0.049 versus untreated cells and *P = 0.04 versus only VEGF-treated cells.

We found that DSGOST inhibited NF-κB signaling which has been revealed to crucially regulate VEGF-dependent angiogenesis. NF-κB-dependent MMP-9 expression is important for endothelial cell movement toward the tumor. Therefore, we further examined whether DSGOST affects NF-κB-dependent MMP-9 expression. When the endothelial cells were transfected with NF-κB reporter plasmid and then treated with VEGF in the presence or absence of DSGOST for 15 hours, DSGOST significantly repressed VEGF-induced transcriptional activity of NF-κB in the luciferase assays (Figure 3A). Consistently, DSGOST reduced expression level of MMP-9 but not CYCLIN D1 (Figure 3B). Moreover, DSGOST decreased MMP-9 activity, when medium from the endothelial cells were subjected to gelatin zymography (Figure 3C). Therefore, DSGOST appears to inhibit NF-κB-dependent MMP-9 expression in VEGF-stimulated endothelial cells.

### DSGOST inhibits vascular leakage *in vivo*

**Figure 4:**
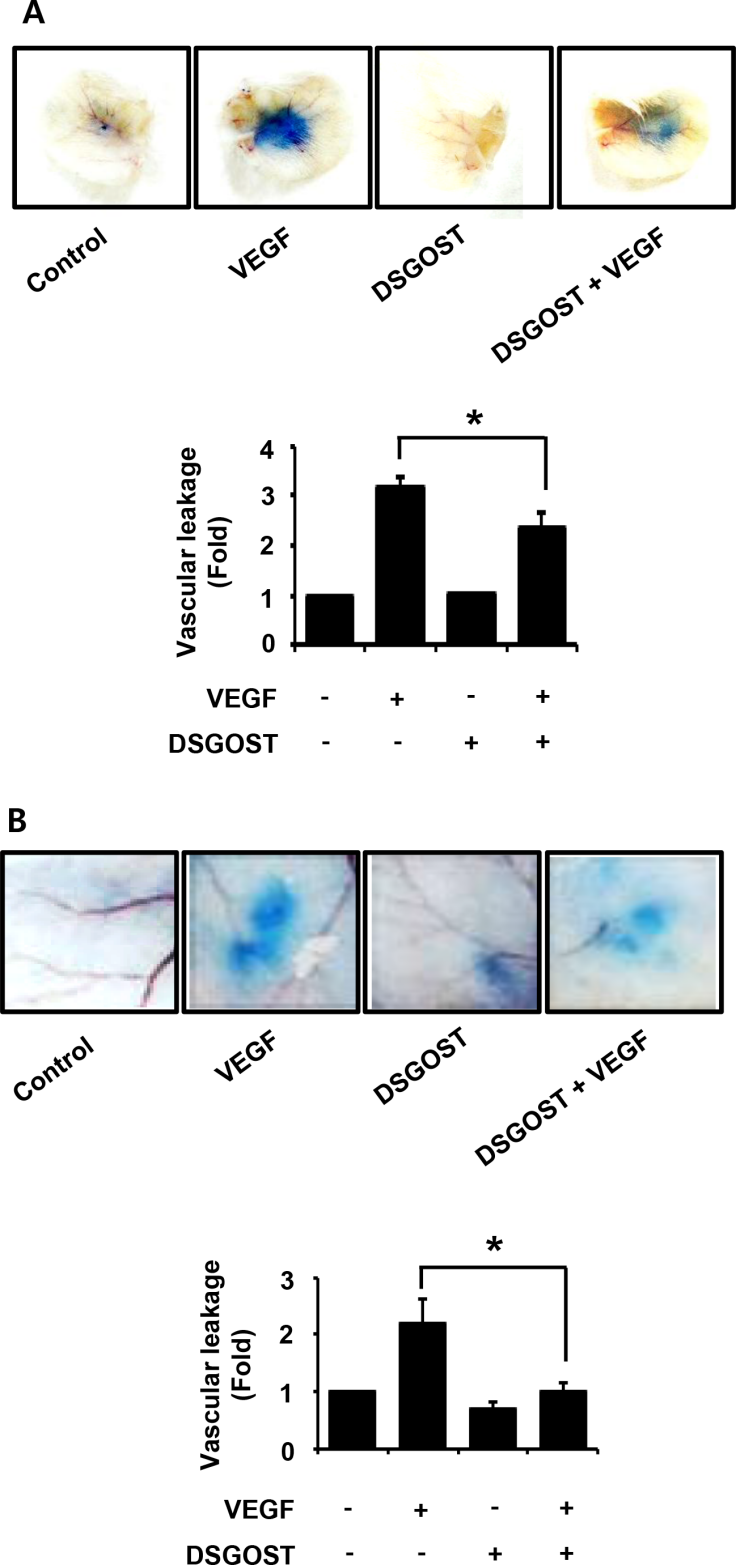
The effect of vascular permeability *in vivo* by DSGOST treatment (**A**) Top, the effect of DSGOST on the vascular permeability of ear was determined by the leakage assay (mean ± SD; *n* = 5). Bottom, data represent quantitative results for left panel. *P = 0.008 versus only VEGF-treated cells (**B**) Top, the effect of DSGOST on the vascular permeability of skin was determined using the leakage assay (mean ± SD; *n* = 5). Bottom, data represent quantitative results for left panel. *P = 0.03 versus only VEGF-treated cells.

As our data showed that DSGOST inhibited VEGF-induced angiogenic abilities of the endothelial cells *in vitro*, we further investigated the anti-angiogenic effect of DSGOST *in vivo*. VEGF induces vascular leakage, which is one of features of angiogenesis. Therefore, we examined whether DSGOST affects VEGF-induced vascular leakage *in vivo*. In mouse ears and back skins, DSGOST reduced VEGF-induced vascular leakages (Figure 4A and 4B). Therefore, our data suggest that DSGOST inhibits VEGF-induced angiogenesis *in vivo*.

### DSGOST inhibits tumor growth *in vivo*

**Figure 5:**
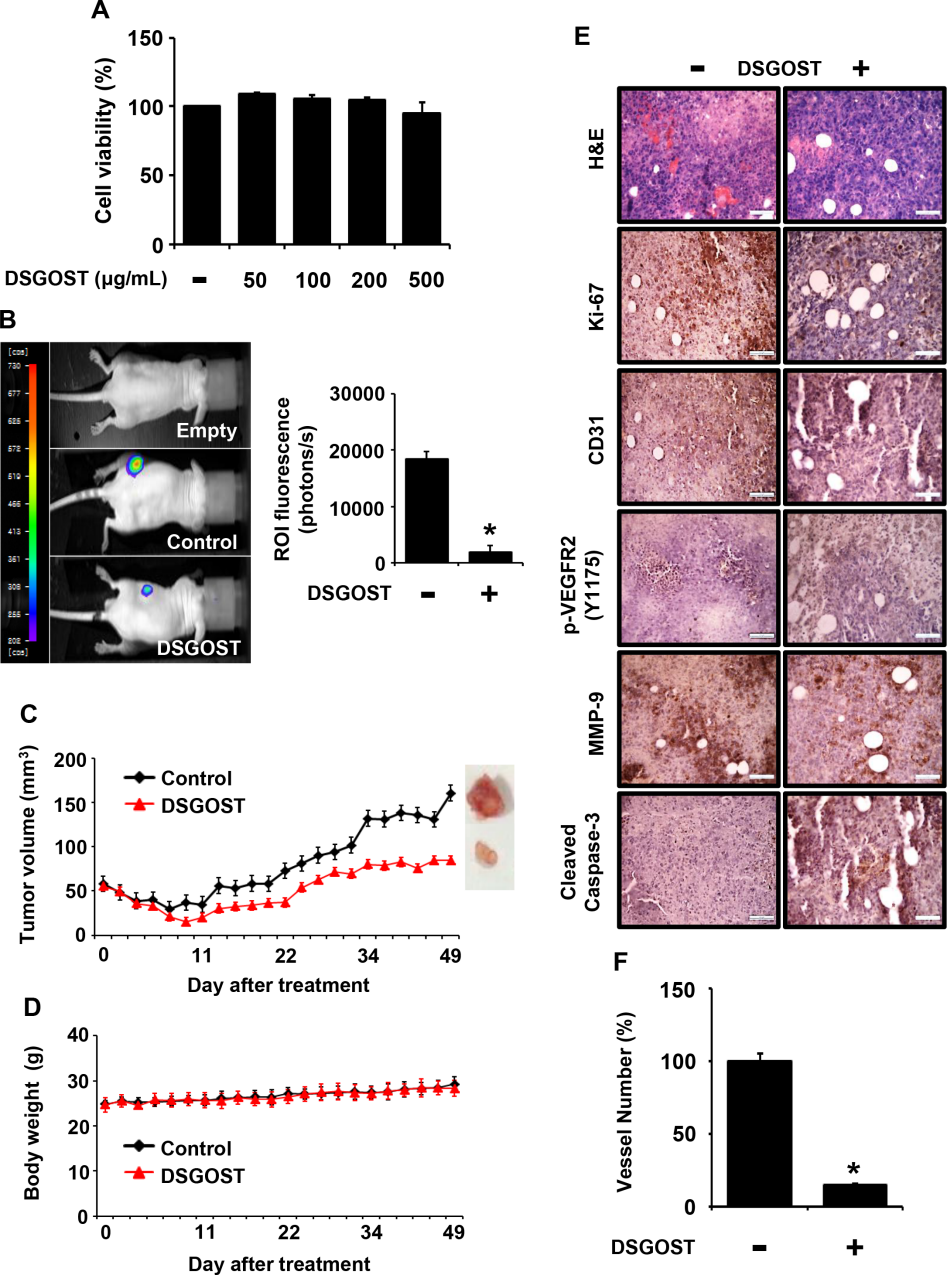
Inhibitory effect of DSGOST on tumor growth and angiogenesis *in vivo* (**A**) The effect of DSGOST on the viability in Panc-28-luc cells was determined by the MTT assay (mean ± SD; *n* = 6). (**B**) Left panel, effects of DSGOST on the growth which tumor derived from Panc-28-luc cell were analyzed by bioluminescence imaging system. Right panel, data represent quantitative results for left panel. (**C-D**) The effect of DSGOST on tumor size or body weight was detected using a caliper or scale. (**E**) Tissue staining for analyzing tumor burden. (**F**) The number of CD31-positive angiogenic vessels (*n*; control group = 7 or DSGOST group = 4). *P = 0.02 versus control group.

It is well known that tumor growth and metastasis require angiogenesis. In addition, VEGF is a crucial environmental cue for tumor angiogenesis. As DSGOST inhibited VEGF-induced angiogenesis both *in vitro* and *in vivo*, we further examined whether DSGOST inhibits tumor growth by targeting tumor angiogenesis. When Panc-28 pancreatic tumor cells were treated with DSGOST at different concentrations, DSGOST did not affect the viability *in vitro* (Figure 5A). When Panc-28-luc cells were *s.c.* injected and then added *p.o.* with DSGOST, the oral administration of DSGOST reduced luciferase-induced *in vivo* bioluminescence, which reflected DSGOST inhibition of tumor growth (Figure 5B). Consistently, DSGOST retarded tumor growth, when tumor volume was measured every second day a week (Figure 5C), while not affecting whole body weight (Figure 5D). Our immunohistochemistry data showed that DSGOST reduced cell numbers stained with anti-phosphorylated Ki67, VEGFR2 or MMP-9 and increased those with anti-cleaved Caspase-3 (Figure 5E), which was consistent with our *in vitro* data. Moreover, DSGOST decreased CD31-stained vessel number in tumor burden (Figure 5E and 5F). Therefore, our data indicate that DSGOST represses tumor growth by inhibiting tumor angiogenesis.

## DISCUSSION

VEGF released from tumor cells activates VEGFR2 expressed in the endothelial cells, which is crucial to drive tumor angiogenesis [4, 6]. Our *in vitro* studies found that DSGOST inhibited VEGF-stimulated migration, invasion and tube formation of the endothelial cells with no effect on the proliferation. Moreover, our *in vivo* data from vascular permeability assays and xenograft mouse tumor growth assays confirmed that DSGOST suppresses VEGF-stimulated angiogenesis. Therefore, our *in vitro* and *in vivo* data strongly suggest the effectiveness of DSGOST on vascular diseases including tumor angiogenesis.

We found that DSGOST inhibited VEGF activation of VEGFR2-mediated signaling pathway including VEGFR2 phosphorylation. While it is yet clearly revealed what chemical components in DSGOST are effective in its anti-angiogenic role, we could postulate effective molecules from the literatures. Cinnamon extract represses VEGF-induced angiogenesis, where procyanidin from cinnamon extract directly inhibits VEGFR2 kinase activity [21]. Likewise, decursin from *Angelica gigas* has been revealed to inhibit VEGF-induced VEGFR2 phosphorylation [22]. Therefore, it was possible that molecules in DSGOST might interrupt the interaction between VEGF and VEGFR2 on the plasma membrane or inhibit VEGFR2 phosphorylation in the cytosol. While it is reported that in HUVECs cinnamon extract may act not only at VEGFR2 but also at VEGFR1 [23], we failed to find DSGOST inhibition of phosphorylation of either VEGFR1 or VEGFR3. Rather, we found that DSGOST directly blocked VEGF binding to VEGFR2. Therefore, it is worth finding herbal compound(s) in DSGOST directly affecting VEGF/VEGFR2 interaction, as our deep knowledge would like to explain a historical effectiveness of TCM and to address scientific notions.

DSGOST inhibition of VEGF/VEGFR2 interaction resulted in the inhibition of various intracellular signaling pathways. We previously found that DSGOST inhibited cold-induced RhoA activation [19]. However, we failed to find DSGOST inhibition of RhoA activation in VEGF-stimulated endothelial cells (data not shown). Thus, it is possible that DSGOST inhibition of intracellular signaling pathways may be dependent on environmental cues.

Our present study concludes that DSGOST inhibits VEGF-induced angiogenesis both *in vitro* and *in vivo*, suggesting that the old established medicine, DSGOST, could be used for a new indication. Our ongoing study will decipher chemical components in DSGOST targeting VEGFR2 activation.

## MATERIALS AND METHODS

### Preparation of DSGOST extracts

Danggui-Sayuk-Ga-Osuyu-Senggang-Tang (DSGOST) was prepared as previously described [19]. In brief, each component was mixed by following recipe; 1 g of *Angelica gigas*, 1 g of *Cinnamomum cassia* Blume, 1 g of *Paeoniae lactiflora* Pallas, 1 g of Akebia root, 0.67 g of Asarum, 0.67 g of *Glycyrrhiza uralensis* Fischer, 1.67 g of *Zizyphus jujube* var. *inermis* Rehder, 0.67 g of Evodia fruit, and 1.33 g of *Zingiber officinale* Rosc. The mixture was extracted by hot water and then was stored at −80°C until use. Information on DSGOST is addressed in Table 1.

**Table 1:** Crude components and amounts of DSGOST

Scientific name	Latin name	Chinese name	Amount (g)
*Angelica gigas*	Angelicae Gigantis Radix	當歸	1.00
*Cinnamomum cassia* Blume	Cinnamomi Ramulus	桂枝	1.00
*Paeonia lactiflora* Pallas (Paeoniaceae)	Paeoniae Radix	芍藥	1.00
Akebia quinata var. polyphylla Nak.	Akebiae Caulis	木通	1.00
Asarum sieboldii var. seoulense Nakai	Asari Herba Cum Radix	細辛	0.67
*Glycyrrhiza uralensis* Fischer (Leguminosae)	Glycyrrhiza Radix	甘草	0.67
*Zizyphus jujuba var. inermis* Rehder	Jujubae Fructus	大棗	1.67
*Evodia* rutaecarpa var. bodinieri Huang	Evodiae Fructus	吳茱萸	0.67
*Zingiber officinale* Rosc.	Zingiberis Rhizoma Praeparata	生薑	1.33

### Cell cultures

Human umbilical vein endothelial cells (HUVECs) were kindly provided by Dr. Kwang Seok Kim (Korea Institute of Radiological and Medical Sciences, Seoul, Korea) and cultured in the endothelial medium supplemented with 5% fetal bovine serum, 1% endothelial cell growth supplement, and 1% penicillin/streptomycin solution. Panc-28-luc cells were kindly provided by Dr. Bharat B. Aggarwal (UT-MDA, Houston, USA) and cultured in DMEM supplemented with 10% fetal bovine serum and 1% penicillin/streptomycin solution.

### Proliferation, wound healing, tube formation, and invasion assay

Either 5 × 10^3^ HUVEC or Panc-28-luc cells were seeded in 96 well plates and then treated with DSGOST at different concentrations. After incubation for 72 hours, the cell viability was measured in MTT colorimetric assays with an absorbance at 570 nm. For wound healing assay, HUVECs were cultured in 12 well plates and then scratched. After treatment with DSGOST and VEGF (50 ng/ml) for 9.5 hours, migrated cells were counted. For *in vitro* tube formation assay, 8 × 10^4^ HUVECs were plated onto 12 well plates, and treated with VEGF (50 ng/ml) and DSGOST at different concentrations. 9 hours after incubation, cells were fixed with 4% paraformaldehyde. Tubule-like structures were then measured. For invasion assays, 6 × 10^4^ HUVECs were plated onto matrigel-precoated 8 μm pore transwell chambers, and the bottom wells were filled with VEGF (50 ng/ml). DSGOST at different concentrations was added onto the upper chambers. Cells were fixed with 4% paraformaldehyde and then stained with 0.05% crystal violet. Invaded cells stained with crystal violet solution were counted to measure the invasiveness. All experiments were performed in triplicate, and then repeated three times, independently.

### Western blot

20 μg of protein was separated by 10∼15% SDS-PAGE and then transferred to nitrocellulose membrane. Appropriate antibodies were used as follows: p-VEGFR2 (Y1175) (#2478), VEGFR2 (#2479), p-FAK (T397) (#3281), p-SRC (Y416) (#2101), p-SRC (Y527) (#2105), SRC (#2109), p-AKT (S473) (#9271), AKT (#9272), JNK (#3708), p-IKKα/β (#9936), IKKα/β (#9936), p-IκBα (#9246), p-NF-κB (#3033), MMP-9 (#3852), and COX-2 (#4842) antibodies were purchased from Cell Signaling (Danver, MA, USA). p-ERK1/2 (sc7383), ERK2 (sc1647), p-JNK (sc6254), NF-κB (sc8008), BCL-2 (c7382), and Cyclin D1 (sc2978) antibodies were from Santa Cruz Biotechnology (Santa Cruz, CA, USA). IκBα (06–494) antibody was obtained Millipore. α-tubulin (T5168) antibody was from Sigma (Thief River Falls, MN, USA). Phospho-VEGFR2 (Y1214) (AF1766) antibody was from R & D systems (Minneapolis, MN, USA). All antibodies were dilutions at 1:1000, tubulin was only dilution at 1:100000.

### Luciferase assay

pNF-κB luciferase reporter vector and pRL-TK luciferase reporter vector as an internal control (Promega, Madison, WI, USA) were co-transfected into the cells using Lipofectamine^®^ 2000 reagent (Invitrogen, NY, USA), when the cells grown as much as 80% confluence. Twenty four hours after transfection, cells at 5 × 10^4^ were transferred in 24 well plates. Next day, cells in each well were treated with 50 ng/ml of VEGF and DSGOST at different concentrations. After 15 hours, luciferase activities were measured using the Dual-Luciferase Reporter Assay System (Promega, Madison, WI, USA) in the luminometer 20/20 n (Turner Biosystems, Sunnyvale CA, USA).

### *In vitro* solid-phase binding assay of biotinylated VEGF to recombinant human VEGFR1–2

The method was performed as described previously [24]. 96-well microplate (Thermo Fisher Scientific, Waltham, USA) was coated with 100 μl of phosphate buffer saline (PBS) containing 500 ng/ml of either VEGFR-1 or -2 ECD/Fc chimera (R & D Systems, Minneapolis, USA). The plate was sealed and incubated overnight at 4°C. After 3 times washes with 200 μl of PBS containing 0.05% (v/v) Tween 20, the plate was blocked by adding 100 μl of PBS with 1% (w/v) bovine serum albumin (BSA), and incubated for 2–3 hours at room temperature. The plate was washed 3 times and added with 100 μl of diluted standards (biotinylated VEGF (btVEGF), R & D systems, Minneapolis, USA) or compounds (with 50 ng/ml btVEGF) in PBS. After 2.5–3 hours incubation at room temperature, the plate was washed 3 times, and 100 μl of streptavidin-HRP (R & D Systems, Minneapolis, USA) was diluted at 1:250 in blocking buffer. The plate was incubated for another 1 hour at room temperature, and then washed five times with 200 μl of wash buffer with 100 μl of substrate solution (BD Biosciences, Sandiego, USA). After 1–3 hours incubation at room temperature, the plate was then added with 50 μl of stop solution (1M H_3_PO_4_) to each well. The signal measured at 450 nm using ELISA plate reader.

### Zymography assay

Cells were treated with VEGF in the presence or absence of DSGOST for 24 hours and then the medium was collected. Medium was concentrated using Amicon Ultra-4 centrifugal filters (Millipore). The concentrated was mixed with non-reducing 5× sample buffer and then loaded directly onto 8% SDS-PAGE containing 0.2% gelatin. Gels were run at 90 V for 3 hours at 4°C and washed for 40 minutes in 2.5% Triton X-100 solution at room temperature. The gels were then incubated in the incubation buffer (50 mM Tris-HCl, 0.15 M NaCl, 10 mM CaCl_2_, pH 7.8) for 20 hours at 37°C, stained with 0.05% Coomassie Brilliant Blue solution for 1 hour, and de-stained until clear bands were visible.

### *In vivo* studies

All *in vivo* experimental procedures were approved by Kyung Hee University Institutional Animal Care and Use Committee (KHU-IACUC). Five-week-old Balb/c and nude mice were purchased from Jungang Lab Animal Inc. (Korea). For vascular permeability assays, VEGF (100 ng/μl) was intradermally injected into the ear or skin in the presence or absence of DSGOST (100 μg). After incubation for 30 minutes, Evans blue dye (30 mg/kg) was injected via tail vein to detect VEGF-induced vascular leakage. The stained tissues were dissected and incubated in formamide solution for 24 hours at 55°C. The extracted dye was measured by colorimetric assay at absorbance with 610 nm. For xenograft mouse tumor growth assays, Panc-28-luc cells (1 × 10^6^) mixed with the matrigels were subcutaneously injected. For the *in vivo* imaging analyses, mice were randomly divided into three groups (background, control, and DSGOST). Saline or DSGOST (20 mg/kg) was orally administrated to the control and DSGOST groups, respectively. Mice were injected with 200 μl D-luciferin using 25 G syringes and incubated for 60 minutes. The image was captured in NightOWL LB 983 and analyzed using Indigo program (Berthold Technologies, Bad Wildbad, Germany). Tumor volume was measured every third day using a caliper, and then determined with the formula as follow: volume = length × width^2^ × 0.5. After 49 days, mice were sacrificed and then tumors were isolated. The tumors were fixed with 4% paraformaldehyde and embedded in paraffin for histological analyses. Immunohistochemistry (IHC) was performed using anti-Ki-67, -CD31, -p-VEGFR2 (Y1175) and -cleaved caspase-3 antibodies.

### Statistics

All experimental data were presented as the mean ± standard deviation, and analyzed by Student *t*-test or one-way ANOVA using SPSS software. *P* value less than 0.05 was considered statistically significant.
